# Relating tissue/organ energy expenditure to metabolic fluxes in mouse and human: experimental data integrated with mathematical modeling

**DOI:** 10.14814/phy2.12159

**Published:** 2014-09-28

**Authors:** China M. Kummitha, Satish C. Kalhan, Gerald M. Saidel, Nicola Lai

**Affiliations:** 1 Department of Biomedical Engineering, Case Western Reserve University, Cleveland, Ohio, USA; 2 Department of Pathobiology, Cleveland Clinic, Lerner Research Institute, Cleveland, Ohio, USA; 3 Department of Pediatrics, Case Western Reserve University, Cleveland, Ohio, USA

**Keywords:** Energy metabolism, flux balance analysis, metabolic pathway fluxes, oxygen consumption, substrate utilization

## Abstract

Mouse models of human diseases are used to study the metabolic and physiological processes leading to altered whole‐body energy expenditure (EE), which is the sum of EE of all body organs and tissues. Isotopic techniques, arterio‐venous difference of substrates, oxygen, and blood flow measurements can provide essential information to quantify tissue/organ EE and substrate oxidation. To complement and integrate experimental data, quantitative mathematical model analyses have been applied in the design of experiments and evaluation of metabolic fluxes. In this study, a method is presented to quantify the energy expenditure of the main mouse organs using metabolic flux measurements. The metabolic fluxes and substrate utilization of the main metabolic pathways of energy metabolism in the mouse tissue/organ systems and the whole body are quantified using a mathematical model based on mass and energy balances. The model is composed of six organ/tissue compartments: brain, heart, liver, gastrointestinal tract, muscle, and adipose tissue. Each tissue/organ is described with a distinct system of metabolic reactions. This model quantifies metabolic and energetic characteristics of mice under overnight fasting conditions. The steady‐state mass balances of metabolites and energy balances of carbohydrate and fat are integrated with available experimental data to calculate metabolic fluxes, substrate utilization, and oxygen consumption in each tissue/organ. The model serves as a paradigm for designing experiments with the minimal reliable measurements necessary to quantify tissue/organs fluxes and to quantify the contributions of tissue/organ EE to whole‐body EE that cannot be easily determined currently.

## Abstract (precis)

e12159

The model relates metabolic flux measurements to the energy expenditure in tissue–organ systems and whole body. The model can be used in designing experiments by selecting the minimal reliable tissue/organs flux measurements necessary to quantify the contributions of tissue/organ to whole‐body energy expenditure that cannot be easily obtained with the current experimental methodologies.

## Introduction

### Mouse‐human metabolism relation

Mouse models are valuable tools to investigate and identify metabolic processes that regulate energy metabolism and body weight (BW) (Tam et al. 2009; Guo and Hall 2011). The results obtained from the models in mice can be translated to humans to a large extent because mice and humans share similar physiological functions at cellular, tissue/organ, and whole‐body levels (Rangarajan and Weinberg 2003; Shultz et al. 2007). However, subtle yet important distinctions are evident in the energy metabolism of mice and humans. For example, the energy expenditure (EE) per gram of body weight in mice is seven times higher than that in humans (Blaxter 1989; Wang et al. 2012) and EE per unit mass of liver and brain is respectively eight times and three times higher in mice than that in humans (Wang et al. 2012). Even though mice and humans share metabolic similarities associated with energy metabolism, the magnitude of these processes in organs and tissues differ significantly between them. Thus, it is important to identify and quantify the metabolic processes that lead to those distinctions in mice and humans for translational research of metabolic diseases. Because key metabolic data are limited and difficult to obtain, modeling is necessary to identify and quantify the metabolic processes involving mice models of human disease.

### Significance of altered metabolic fluxes

Fuel homeostasis in the whole body requires coordination of metabolic fluxes among organs and tissues. These are regulated by neuroendocrine and hormonal factors (Hall 2006; Kim et al. 2007; Pattaranit and van den Berg 2008). The whole‐body metabolic fluxes that are important for energy metabolism are glycolysis, glycogenolysis, gluconeogenesis, lipolysis, *de novo* lipogenesis, triglyceride‐fatty acid cycling, proteolysis, and oxidation of macronutrients (carbohydrate, fat, and protein). The total EE is equal to sum of the rates of oxidation of macronutrients. These fluxes change in chronic disease (e.g., diabetes), exercise and dietary perturbations as a result of altered cellular metabolic processes in various tissues and organs (Hall 2006; Kim et al. 2007; Pattaranit and van den Berg 2008). These pathophysiologic perturbations alter metabolic pathways and fluxes in individual organs and alter interorgan exchange rates of substrates with subsequent changes in substrate utilization, EE, and BW (Hall 2006; Kim et al. 2007; Pattaranit and van den Berg 2008). Although most metabolic pathways of substrate utilization are known, the relationships between these pathways and body weight regulation are yet to be quantified. By quantifying EE and metabolic pathway fluxes in organs and tissues, we can obtain key information that relates changes in metabolic processes with regulation of energy metabolism and BW in disease.

### EE and metabolic fluxes

Several techniques are available to measure organ/tissue EE and metabolic fluxes in animal models and humans. The product of blood flow and arterio‐venous difference of oxygen is commonly used to determine organ/tissue oxygen consumption (VO_2_) in vivo. The VO_2_ of different organs/tissues is then used to quantify their contribution to the whole‐body EE (Elia 1992). The application of this approach is limited in mice because it is challenging to measure blood flow and arterio‐venous difference of oxygen across organs/tissues. Alternatively, investigators have used allometric equations of EE and BW to obtain organ EE in animals (Wang et al. 2012). This approach does not account for changes in body composition and its contribution to whole‐body energy metabolism. Stable isotope tracers combined with measurements of isotopomer labeling using NMR and mass spectroscopy are used to determine metabolic pathway fluxes in vivo (Choi and Antoniewicz 2011). However, they require fairly large amount of sample, long analytical time, and expensive equipment and provide partial information about the distribution of isotopomers. Thus, it is desirable to identify the minimal number of the metabolic flux measurements required to quantify the energy metabolism of each organ in relation to the whole‐body energy expenditure.

### Mathematical models of energy metabolism

To relate energy metabolism to the regulation of BW in humans and mice, mathematical models have been developed (Hall 2006, 2012; Tam et al. 2009; Guo and Hall 2011). Although these models can identify whole‐body metabolic fluxes responsible for changes in body weight and composition in response to dietary changes, they do not quantify the metabolic processes in the organs responsible for body weight regulation. Previously, a complementary approach was developed to evaluate metabolic fluxes of organs and tissues by integrating stoichiometric metabolic network models with organ/tissue measurements of uptake and/or production of metabolites and metabolic fluxes (Kim et al. 2007, 2011; Li et al. 2009). By this method, in vivo fluxes can be quantified and relate metabolism of organs and tissues to whole‐body at rest and during exercise in humans. In this study a similar mathematical approach is applied to quantify organ/tissue metabolic fluxes in mice.

Here, we develop a unique quantitative framework to estimate metabolic fluxes of the main pathways of energy metabolism in key tissue/organs of the mouse. Our mathematical framework integrates mass balances, energy balances, and metabolic fluxes obtained from the literature. Specific assumptions are also used to estimate metabolic fluxes that are difficult to measure in each organ of the mouse and are not available in literature. Consequently, the model is used to evaluate (1) the metabolic pathway fluxes of tissues and organs from a limited set of experimental data and (2) the contribution of tissue/organ energy metabolism to whole‐body energy metabolism. Furthermore, by quantifying differences of whole‐body and intraorgan metabolic fluxes between mouse and human, we could relate energy metabolism of mice to humans.

## Methods

### Overview

In this work, a model paradigm is developed to relate organ‐level energy expenditure to metabolic flux as an alternative to the Fick principle in mice. The main goal is to provide a method to quantify the energy expenditure of organs using metabolic fluxes of the main pathways involved in fuel metabolism. Here, the main organs and tissues involved in lipid, carbohydrates, and protein metabolism and the organs for which there is sufficient metabolic information about mice are considered. The methodology presented here allows quantitative analysis of metabolic fluxes (MF) of overnight‐fasted mouse organs/tissues: brain, heart, liver, skeletal muscle, adipose tissue, and gastrointestinal tract (GI), which includes stomach, spleen, intestines, and visceral fat. The model provides a mechanistic framework to study substrate utilization in each organ. Liver, gastrointestinal (GI) tract, skeletal muscle, and adipose tissue are key organs/tissues that contribute to the adaptive responses to pathophysiological conditions and provide metabolic fuels necessary for sustenance. Additionally, brain and heart consume energy for sending biochemical signals and transport energy. Because of insufficient data on fuel metabolism of lung and kidney in mice, these organs are not included.

**Figure 1.**
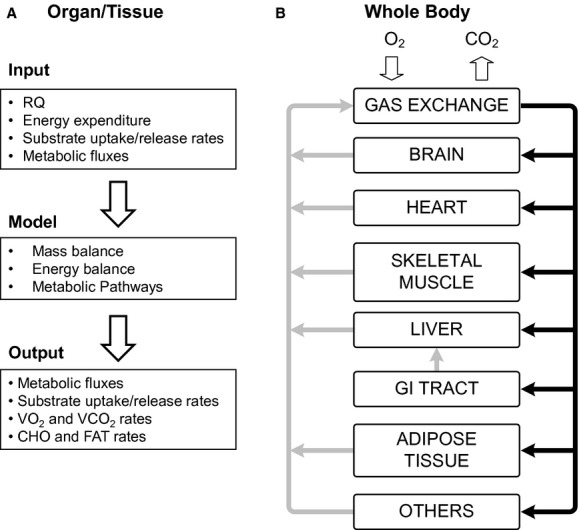
(A) Essential model inputs and equations for estimating computational outputs; (B) Whole‐body systems:Venous (gray arrows) and arterial blood (black arrows) leaving/going to the organ/tissue systems, respectively. RQ is respiratory quotient; VO_2_ and VCO_2_ are oxygen consumption and carbon dioxide release rates respectively; CHO and FAT are rates of carbohydrate and fat utilization.

Steady‐state mass balance equations are developed for each key metabolite in the biochemical pathways of organs and tissues. This builds upon the approach by others (Kim et al. 2007) used to determine organ/tissue MFs of humans. For mice, however, data are lacking in regard to rates of substrate uptake/release and MFs to construct all the pathway fluxes of organs/tissues. To compensate for this lack of data, the mathematical model combines mass and energy balances to quantify organ/tissue energy expenditure (EE). Consequently, this model analysis yields MFs, substrate uptake/release, substrate utilization, oxygen consumption (VO_2_), and carbon dioxide production (VCO_2_) in various organs/tissues of mice. The data inputs given in Tables 1–4 and 10 allow the mathematical model (Fig. 1) to predict the data outputs (Table 5–Table 9 and 12).

**Table 1.** Metabolic fluxes (MFs) of mouse organs/tissues.

Organ/tissue	MF	(*μ*mol/min/kg)^1^	Reference
Brain	*ϕ*_GLY→G6P_	2.0	(Kim et al. 2007)
	*ϕ*_PYR→LAC_	469.8	(Kim et al. 2007)
Heart	*ϕ*_GLY→G6P_	160.0	(Kim et al. 2007)
	*ϕ*_PYR→LAC_	352.0	(Kim et al. 2007)
	*ϕ*_TG→GLR_	16.0	(Kim et al. 2007)
Liver	*ϕ*_GLC→G6P_	73.1^2^	(Mulligan and Tisdale 1991)
	*ϕ*_G6P→GAP_	73.1^3^	(Kim et al. 2007)
	*ϕ*_GAP→PYR_	146.2^3^	(Kim et al. 2007)
	*ϕ*_PYR→LAC_	140.0	(Kim et al. 2007)
	*ϕ*_G6P→GLY_	66.0	(Kim et al. 2007)
	*ϕ*_GLY→G6P_	305.4^2^	(Chacko et al. 2012)
	*ϕ*_TG→GLR_	2.7	(Kim et al. 2007)
	*ϕ*_AcoA→FFA_	74.7	(Kim et al. 2007)
	*ϕ*_PYR→ACoA_	0.0	(Kim et al. 2007)
GI	*ϕ*_PYR→LAC_	100.0	(Kim et al. 2007)
Skeletal muscle	*ϕ*_LAC→PYR_	44.4	(Kim et al. 2007)
	*ϕ*_GLY→G6P_	6.2	(Kim et al. 2007)
	*ϕ*_TG→GLR_	6.5	(Kim et al. 2007)
Adipose tissue	*ϕ*_PYR→LAC_	3.3	(Kim et al. 2007)
	*ϕ*_LAC→PYR_	0.9	(Kim et al. 2007)
	*ϕ*_FFA→TG_	138.4^4^	(Kim et al. 2007)

All fluxes otherwise indicated by ^2^, ^3^ and ^4^ are calculated using the assumption that the metabolic fluxes (MFs) (per unit organ/tissue mass) in mouse and human are similar.

The *ϕ*_GLC→G6P_ and *ϕ*_GLY→G6P_ in the mouse liver were obtained using isotope tracers.

^1^per kg of organ weight.

^2^Experimental data.

^3^The relationships of MFs for *ϕ*_G6P→GAP_ (=*ϕ*_GLC→G6P_) and *ϕ*_GAP→PYR_ *(=2xϕ*_GLC→G6P_) in mouse liver were based on the fluxes in human liver.

^4^The relationship of MFs for *ϕ*_FFA→TG_ (=0.2*ϕ*_TG→FFA_) in mouse adipose tissue was based on the fluxes in human adipose tissue. 20% of the FFA resulted from lipolysis reesterified to TG.

**Table 2.** Mouse organ/tissue substrate uptake/release rates.

Organ/tissue	Uptake (Upt) Release (Rel)	Uptake/Release as %*R*_*a*_ of substrate	Upt/Rel (*μ*mol/min/kg)^1^	Reference
Heart	Upt_LAC_	12.9% of *R*_*a*,LAC_	708.3	(Kim et al. 2007)
Liver	Rel_GLC_	100% *R*_*a*,GLC_	1154.8^2^	(Chacko et al. 2012)
	Rel_TG_	100% *R*_*a*,TG_	10.8	(Kim et al. 2007)
	Upt_GLR_	100% of *R*_*a*,GLR_	545.7	(Kim et al. 2007)
	Upt_ALA_	100% of *R*_*a*,ALA_	860.2	(Kim et al. 2007)
GI	Upt_TG_	20.7% of *R*_*a*,TG_	1.6	(Kim et al. 2007)
	Rel_FFA_	36.2% of *R*_*a*,FFA_	353.3	(Kim et al. 2007)
Skeletal muscle	Upt_GLC_	Mouse data	58.3^2^	(Toyoda et al. 2011)
	Rel_LAC_	36.1% of *R*_*a*,LAC_	32.8	(Kim et al. 2007)
	Upt_TG_	10.3% of *R*_*a*,TG_	0.2	(Kim et al. 2007)
	Rel_GLR_	–	0.2^3^	(Kim et al. 2007)
	Rel_ALA_	100% of *R*_*a*,ALA_	155.8	(Kim et al. 2007)
Adipose tissue	Upt_TG_	69% of *R*_*a*,TG_	4.5	(Kim et al. 2007)
	Rel_GLR_	70.4% of *R*_*a*,GLR_	230.6	(Kim et al. 2007)
	Rel_FFA_	63.7% of *R*_*a*,FFA_	531.2	(Kim et al. 2007)

*R*_*a*_, appearance rate; Upt, substrate uptake; Rel, substrate release rate.

All substrate uptake/release rates otherwise indicated by ^2^ and ^3^ are calculated using the following assumption. The appearance rate fraction of metabolic fuels taken out (or released) of (or into) plasma by organs/tissues is similar in both human and mouse. Mouse organ/tissue substrate uptake/release rates were calculated by multiplying appearance rate fraction of metabolic fuels of human organs/tissues with mouse appearance rate of substrates in plasma (R_*a*,*i*_) reported in Table 10.

The Rel_GLC_ from liver and Upt_GLC_ into skeletal muscle were determined using isotope tracers.

^1^Per kg of organ weight.

^2^Experimental data.

^3^The relationships of Rel_GLR_ = Upt_TG_ in mouse muscle was based on the substrate uptake/release rate in human muscle.

**Table 3.** Mouse and human physiological parameters.

Organ/Tissue	Mass				Blood flow		Respiratory quotient (RQ) (Kim et al. 2007)^3^
	Mouse (Martin and Fuhrman 1955)		Human (Lindstedt and Schaeffer 2002; Kim et al. 2007)		Mouse (Fenneteau et al. 2009)	Human (Kim et al. 2007)	Mouse/Human
	(g)	(% of BW)	(10^3^g)	(% of BW)	(mL/min/100 g)^1^	(mL/min/100 g)^1^	(−)
Brain	0.54	1.8	1.49	2.1	98.15	50.34	1.0
Heart	0.17	0.57	0.25	0.36	658.82	100.0	0.79
Liver	1.86	6.2	1.5	2.1	146.77	100.0	0.72
GI tract	2.65	8.83	2.0	2.9	90.19	55.0	1.0
Skeletal Muscle	10.27	34.23	27.8	39.7	26.19	3.24	0.78
Adipose Tissue	3.10	10.33	11.0	15.7	38.39	3.27	0.81
Others	11.41	38.03	25.96	37.1	55.21	2.47	0.80/0.67 (this work)
Whole body	30.0	100.0	70.0	100.0	56.47	7.86	0.77/0.8^2^

We assumed that mouse and human organs have same RQ.

^1^Per 100g of organ weight.

^2^Mouse whole‐body RQ is 0.77 (Kaiyala et al. 2010) and human whole‐body RQ is 0.8 (Kim et al. 2007).

^3^Mouse and or human.

**Table 4.** EE of mouse and human organs/tissues.

Organ/Tissue	Mouse			Human			Mouse/Human
	(kcal/kg/day)^1^	(kcal/day)	(%)	(kcal/kg/day)^1^	(kcal/day)	(%)	*X*_*i*_‐fold
Brain	740.7	0.4	6.42	247.1	368.2	21.35	3.0
Heart	1352.9	0.23	3.69	705.5	176.4	10.23	1.92
Liver	1747.3	3.25	52.17	224.5	336.7	19.53	7.78
GI tract	52.8	0.14	2.25	36.8	73.6	4.27	1.43
Skeletal muscle	78.9	0.81	13.0	13.0	361.9	20.99	6.06
Adipose tissue	100.0	0.31	4.98	4.1	44.9	2.6	24.5
Others	95.6	1.09	17.5	14	362.7	21.03	6.84
Whole body	207.7	6.23	100.0	24.6	1724.4	100.0	8.43

EE of brain, heart, liver were determined using allometric equations that relate organ/tissue EE to body mass. The EE of GI tract and “others” were obtained using “residual organs” allometric equation (Wang et al. 2012). “Others” includes the rest of the organs/tissues including kidneys. Adipose tissue EE was determined from FM EE (Guo and Hall 2011). Muscle EE was determined by subtracting brain, heart, liver, GI tract, and others EE from FFM EE (Guo and Hall 2011; Wang et al. 2012).

Human organ/tissue EE was determined from sum of carbohydrate and fat utilization rates (Kim et al. 2007).

*X*_*i*_‐fold for each organ/tissue: mouse EE (kcal/kg/day)/human EE (kcal/kg/day).

^1^Per kg of organ weight.

**Table 5.** Mouse and human organ/tissue substrate uptake/release rates.

Organ/Tissue	Substrate	Substrate Uptake/Release (*μ*mol/min/kg)^1^		
		Mouse		Human
		Calculated	Measured	Calculated/Measured
Brain	Glucose	764.4	1270.0, 700.0 (Growdon et al. 1971; Mulligan and Tisdale 1991)	255.0
Heart	Glucose	108.7	49.1 (Matsui et al. 2006)	160.0
	Free fatty acid	268.8	NA	140.0
Liver	Free fatty acid	592.8	NA	140.0
	Lactate	437.1	NA	180.0
GI tract	Glucose	54.5	236 (Mulligan and Tisdale 1991)	38.0
	Glycerol	−117.8^2^	NA	−20.0^2^
Skeletal muscle	Free fatty acid	13.6	NA	2.3
Adipose tissue	Glucose	59.1	43 (Mulligan and Tisdale 1991)	3.50
	Lactate	−2.4^2^	NA	5.1

NA, not available.

^1^Per kg of organ weight.

^2^The negative sign indicates substrate release.

**Table 6.** Mouse VO_2_ and VCO_2_ rates calculated with flux balance analysis (FBA) and standard approach.

Organ/Tissue	VO_2_ (mL/min/kg^1^)		VCO_2_ (mL/min/kg^1^)	
	FBA	Standard approach	FBA	Standard approach
Brain	102.74	102.47	102.74	102.47
Heart	200.70	197.40	158.55	155.95
Liver	278.19	259.68	200.29	186.97
GI tract	7.33	7.31	7.33	7.31
Skeletal muscle	11.45	11.54	8.93	9.00
Adipose tissue	15.69	14.51	12.71	11.76

^1^Per kg of organ weight.

**Table 7.** Whole‐body fuel metabolic fluxes.

Metabolic flux	Mouse	Human	*X*_*i*_‐fold
	(*μ*mol/min/kg)^1^		Mouse/Human
Glycogenolysis	14.8	5.4	2.7
Gluconeogenesis	56.8	5.0	11.4
De novo lipogenesis	4.8	0.3	16.7
Proteolysis	44.3	4.0	11.1
Lipolysis	36.7	3.9	9.4

*X*_*i*_‐fold for each flux: mouse flux/human flux.

^1^Per kg of body weight.

**Table 8.** Mouse organ/tissue metabolic fluxes.

Fluxes	Metabolic fluxes (*μ*mol/min/kg)^1^					
	Brain	Heart	Liver	GI	Skeletal muscle	Adipose tissue
GLC *→* G6P	764.4	108.7	**73.1**	54.5	58.3	59.3
G6P *→* GAP	764.4	108.7	**73.1**	54.5	58.3	59.3
GAP *→* PYR	1528.8	217.5	**146.2**	109.0	116.6	72.5
PYR *→* GAP	–	–	1443.6	–	–	–
GAP *→* G6P	–	–	1978.5	–	–	–
G6P *→* GLC	–	–	1228	–	–	–
G6P *→* GLY	2.0	160.0	**66.7**	–	6.25	–
GLY *→* G6P	**2.0**	**160.0**	**305.4**	–	**6.25**	–
PYR *→* LAC	**469.8**	**352.0**	**140.0**	**100.0**	77.2	**3.3**
LAC *→* PYR	469.8	1060.3	577.1	100.0	**44.4**	**0.9**
GLR *→* GRP	–	16.0	548.4	–	6.3	0.0
GAP *→* GRP	–	–	0.0	–	0.0	46.1
GRP *→* GAP	–	–	534.9	–	0.0	–
PYR *→* ALA	–	–	0.0	–	26.5	–
ALA *→* PYR	–	–	860.2	–	0.00	–
PYR *→* ACoA	1528.8	925.8	**0.0**	109.0	57.3	70.2
FFA *→* ACoA	–	268.8	569.7	–	14.2	22.3
ACoA *→* FFA	–	–	**74.7**	–	–	–
TGL *→* GLR	–	**16.0**	**2.7**	117.8	**6.50**	230.6
FFA *→* TG	–	48.0	40.4	–	18.9	138.4
ACoA *→* CO_2_	1528.8	3076.1	4483.1	109.0	170.7	248.6
O_2_ *→* H_2_O	4586.3	8959.4	12418.8	327.1	511.3	700.5
ATP *→* ADP	30574.1	56241.7	72908.6	2180.5	3276.6	4156.8
Protein *→* ALA	–	–	–	–	129.3	–

Values in bold are assumed fluxes (see Table 1) and the rest are calculated with flux balance analysis.

^1^Per kg of organ weight.

**Table 9.** Carbohydrates and fat oxidation rates in mouse and human organs.

Organ/Tissue	Substrate utilization (*μ*mol/min/kg)^1^				*X*_*i*_‐fold	
	Mouse		Human		Mouse/Human	
	CHO	FAT	CHO	FAT	CHO	FAT
Brain	30574	0	10199	0	3.0	–
Heart	17163	39079	9028	20300	1.9	1.9
Liver	−4251.2	77160	−1700	11078	2.5	7
GI tract	2180.5	0	1520	0	1.4	–
Skeletal muscle	1197.7	2078.9	180.84	360.21	6.6	5.8
Adipose tissue	1282.7	2874.2	52.12	117.52	24.6	24.5

CHO, carbohydrate; FAT, fat.

*X*_*i*_‐fold for each organ/tissue: mouse substrate utilization/human substrate utilization.

Negative sign indicates CHO production.

^1^Per kg of organ weight

### Mathematical model

**Figure 2.**
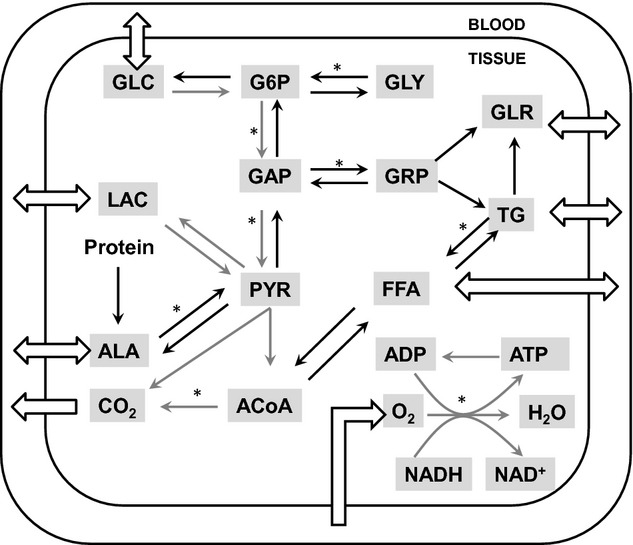
General metabolic pathways in whole‐body model. Eight substrates connected with open arrays are transported between tissue and blood. While gray arrows are common pathways in all tissues, black arrows are tissue‐specific pathways. The pathways marked with (*) are composed of several reaction steps but lumped into one step in this model. ADP, adenosine diphosphate; ATP, adenosine triphosphate; ACoA, acetyl CoA; AA, amino acids; GLC, glucose; G6P, glucose‐6‐phosphate; GAP, glyceraldehyde‐3‐phosphate; GLR, glycerol; GRP, glycerol‐3‐phosphate; GLY, glycogen; FFA, free fatty acid; LAC, lactate; PYR, pyruvate; TG, triglycerides.

**Figure 3.**
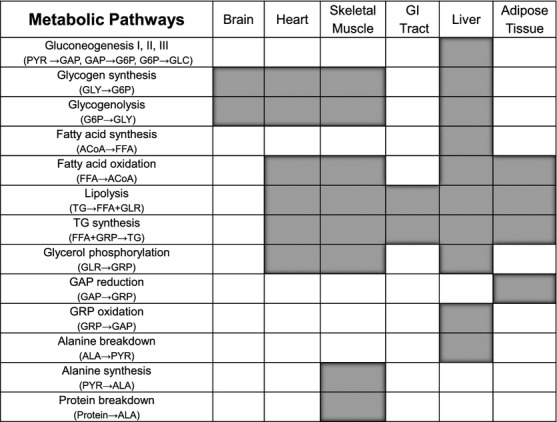
Map for tissue‐specific metabolic pathways. In addition to the common pathways shown in Figure 2, each tissue has different kinds of metabolic pathways. Blank filled with gray color means the existence of the corresponding pathway.

Based on the primary function of the organ/tissue in the whole‐body energy metabolism, we specified the major specialized metabolic pathways, which dictate the exchange and distribution of metabolic fuels among tissues/organs. The main metabolic fuels that exchange among tissues and organs via blood circulation are glucose, free fatty acid, glycerol, triglyceride, lactate, and amino acids (represented here by alanine) (Fig. 2). The systems of metabolic reactions that are present in each tissue and organ are provided in Figure 2 and Appendix 1 (Kim et al. 2007). The distinctive metabolic reactions present in each tissue and organ are shown in Figure 3. The protein breakdown is present in most of the organs/tissues after overnight fasting, however, we considered proteolysis only in skeletal muscle because in all other organs the contribution of proteolysis to whole body is not significant compared to skeletal muscle. Furthermore, although gluconeogenesis also takes place in the GI tract but we neglected for this analysis because its contribution to the whole body is not significant.

#### Mass balances

A system of mass balance equations is defined for each tissue/organ system. The mass balance for each metabolite is based on the metabolic flux (production/utilization) and uptake/release rates of the metabolite in each tissue/organ. The metabolic fluxes of substrate production and utilization in tissues and organs depend on many complex biochemical reactions. We assume that the tissue and capillary subcompartments are spatially lumped in all tissues and organs. The concentration dynamics C_*x*,*i*_(*t*) of each substrate (*i*) in each tissue and organ (*x*) can be described by the following dynamic mass balance equation:
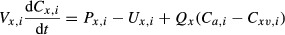


where *V*_*x,i*_ is the volume of substrate *i* in tissue or organ *x*,* P*_*x,i*_, and *U*_*x,i*_ are the substrate production and utilization rates in tissue or organ *x*. *Q*_*x*_ is the tissue or organ blood flow rate. The input arterial concentration is *C*_*a,i*_ and the output venous concentration is *C*_*xv,i*_. At steady state, the transient term is zero so that
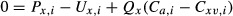


The uptake (Upt_*x,i*_) or release (Rel_*x,i*_) of substrate *i* in tissue or organ x is related to blood flow and arterio‐venous difference:
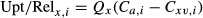


For substrates that exist only within tissues/organs, we set *Q*_*x *_= 0. The net rate of metabolic reaction is
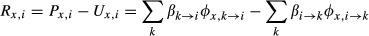


where *ϕ*_*x*,*k*→*i*_ and *β*_*k*→*i*_ are the flux and stoichiometric coefficient of the reaction from substrate *k* to substrate *i*, respectively. The steady‐state mass balance equations for the system of reactions shown in Figure 2 and Appendix 1 are presented in Appendix 2. The specific metabolic functions of each tissue/organ system and the number of metabolites in the pathways determine steady‐state mass balance equations of organs/tissues, which vary from one organ/tissue to another.

#### Energy balances

The EE for each organ and tissue is related to the carbohydrate and fat oxidation according to the following equation:
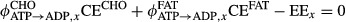


where CE^CHO^ and CE^FAT^ are the calorific ATP equivalents of carbohydrate and fat oxidation, respectively and 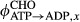 and 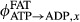 are the carbohydrate and fat oxidation for organ *x* (Appendix 3). These fluxes are calculated according to
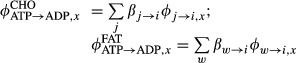


where 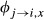, 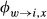 and *β*_*j*→*i*_, *β*_*w*→*i*_ are fluxes and stoichiometric coefficients of the reaction from substrate j (or w) to substrate *i* associated with carbohydrate (or fat) utilization.

We solved coupled steady‐state mass and energy balance equations numerically to obtain estimates of mouse organ/tissue MFs (using MATLAB R2011b, fsolve). We also computed rates of substrate utilization, VO_2_, and VCO_2_ for each organ/tissue from the MFs. A model calculation for estimating liver metabolic fluxes is provided in the Appendix 6. The “others” organs/tissues VO_2_ is determined by subtracting the VO_2_ of brain, heart, liver, GI tract, muscle, and adipose tissue from the whole‐body VO_2_.

We also used standard empirical relationships (Tang et al. 2002) to compute whole‐body and organ/tissue (*x* = brain, liver, heart, skeletal muscle, adipose tissue, GI tract, others) VO_2_:
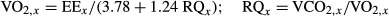


The VO_2_ and VCO_2_ rates (per unit mass of organ/tissue) thus obtained using both FBA and standard approach are compared.

### Model inputs

Substrate uptake/release, metabolic fluxes, energy expenditure, and respiratory quotients are the data inputs to the organ/tissue mathematical model (Fig. 1). To the extent possible, available data from literature are used. In the absence of experimental data, specific assumptions are made to determine metabolic pathway fluxes based on current knowledge of fuel homeostasis in human and mice. Quantification of the key information in each organ/tissue of the mouse is described in the following sections.

#### Mouse physiological parameters

The model analysis is based on a 30 g adult wild‐type mouse. The weights of the organs and tissues were determined from measurements of organ weights expressed as percent of total body weight (Martin and Fuhrman 1955). The rates of organ and tissue blood flow were calculated from blood flow rates expressed as a fraction of the cardiac output (Q) (Fenneteau et al. 2009). The mouse organ and tissue weights and blood flows are reported in Table 3. The respiratory quotient (RQ) of each organ and tissue in mouse is assumed to be the same as that of an overnight fasting human (Kim et al. 2007) (Table 3). This assumption is consistent with the experimental evidence that whole‐body RQ under fasting conditions is similar in both human and mice (Kim et al. 2007; Kaiyala et al. 2010).

#### Appearance rates of substrate in plasma

The appearance (or disappearance) of metabolic fuels in plasma occurs when one or more organs and tissues release (or take up) substrates. Under steady‐state conditions, the appearance rate equals the disappearance rate. The rate of appearance of various substrates in plasma measured in an overnight fasting (8–16 h) mouse from tracer infusion studies are reported in Table 10 (Andrikopoulos and Proietto 1995; Xu et al. 2002; Goudriaan et al. 2005; Bergman et al. 2006; Chacko et al. 2012).

**Table 10.** Appearance rates of metabolic fuels in the plasma of mouse and human.

Metabolic fuel	Appearance rate (*R*_*a*_) (*μ*mol/min/kg)^1^		Appearance rate (*R*_*a*_) (*μ*mol/min)		*X*_*i*_‐fold
	Mouse	Human	Mouse	Human	Mouse/Human
Glucose	71.6 ± 4.57 (Chacko et al. 2012)	10.87	2.15±0.14	761.0	6.59
Lactate	31.12 (this work)	4.43	0.93 (this work)	310.0	7.02
Pyruvate	0.0 (this work)	0.07	0.0 (this work)	5.0	NA
Alanine	64.9 ± 11.8 (Andrikopoulos and Proietto 1995)	4.57	1.95 ± 0.35	320.0	14.21
Free Fatty acid	96.3 ± 17.3 (Bergman et al. 2006)	4.73	2.89 ± 0.52	331.0	20.37
Glycerol	32.6 ± 4.3 (Xu et al. 2002)	2.00	0.98 ± 0.13	140.0	16.30
Triglyceride	0.67 ± 0.03 (Goudriaan et al. 2005)	0.41	0.020 ± 0.001	29.0	1.63

NA, not available.

*X*_*i*_‐fold for each organ/tissue: mouse *R*_*a*_ (*μ*mol/kg/min)/human R_*a*_ (*μ*mol/kg/min). ^1^Per kg of body weight.

The references for mouse substrate appearance rates are reported in the brackets. Human substrate appearance rates were obtained from Kim et al. (2007).

#### Substrate uptake/release rates

The rate of uptake of glucose determined using isotope tracers are available in literature for brain, heart, GI tract, skeletal muscle, and adipose tissue (Tables 2 and 5). Unknown mouse substrate uptake/release rates were calculated based on appearance rates of substrate in the plasma of mice and the fractional rates of substrate uptake/release in humans (Table 2):
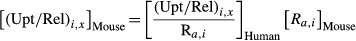


Fractional rates of substrate uptake/release determine tissue/organs contributions to appearance rates of substrate in the plasma. It is assumed that the appearance rate fractions of metabolic fuels taken (or released) out of (or into) plasma by organs/tissues are similar in both human and mouse (Table 2). Based on the literature (Kim et al. 2007), we can specify substrate uptake (or release) by (or from) each tissue and organ. We assume that all the glucose that appears in plasma comes from liver and all other organs/tissues consume glucose, which holds true both in human and mouse. Adipose tissue (AT) and GI tract are the sources of FFA in the plasma, while all other organs consume FFA. Glycerol is released from AT, GI tract, and skeletal muscle (SM), while liver consumes all plasma glycerol. Triglyceride (TG) is released by liver, while TG is consumed by the GI tract, SM, and AT. Alanine is released by SM and consumed by liver. We assumed alanine as the representative amino acid of all amino acids. Lactate is released by SM, AT, and “others” tissues (e.g., red blood cells) and consumed by liver and heart. The organ/tissue substrate uptake/release rates are presented in Tables 2.

#### Metabolic fluxes

The metabolic fluxes (MFs) of glucose to glucose‐6‐phosphate (*ϕ*_GLC→G6P_) and glycogen to glucose‐6‐phosphate (*ϕ*_GLY→G6P_) are available in literature for mouse liver, which are determined using isotope tracers (Table 1). For any MF that is not known from literature, we assume organ/tissue MFs that the reaction flux (per unit weight of organ/tissue) in mouse is equal to the reaction flux in human (Table 1). This assumption corresponds to the flux relationship between *ϕ*_GLC→G6P_ and *ϕ*_GLY→G6P_ from MFs measured in mouse and human liver (Mulligan and Tisdale 1991; Kim et al. 2007; Chacko et al. 2012).

#### Organ energy expenditure

To calculate EE of mouse organs and tissues, we used an allometric function that relates EE (kcal/kg/day) to body mass BW (kg) (Wang et al. 2012):
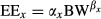


where *α*_x_ and *β*_x_ are the parameters for organ *x*, which are reported in Table 11. EE_x_ refers to the energy expenditure of organs and tissues, and BW refers to the mouse whole body mass under overnight fasting conditions (unless otherwise specified). Similar allometric functions that relate organ size to body mass were successfully used to estimate organ masses of different mature mammalian species ranging in body size from mice to elephants (Elia 1992; Wang et al. 2001). Furthermore, the whole‐body EE determined from the sum of the EE of individual organs predicted the whole‐body EE, which is a function of BW (Wang et al. 2012). Therefore, we chose this relation as a first approximation to obtain mouse organ/tissue EE.

**Table 11.** Parameters of the organ/tissues EE allometric relationships (Wang et al. 2012).

Organ/Tissue	*α*	*β*
Brain	446.6	−0.1423
Heart	890.3	−0.1181
Liver	683.9	−0.2677
Kidneys	689.7	−0.0833
Other organs	29.96	−0.1667

This allometric equation was used to calculate EE only for brain, heart, liver, kidney, and “residual” organs/tissues. The EE of the GI tract was evaluated using equation 9 with the parameters of the “residual organs”. The EE of “others” was evaluated as the weighted average of kidney EE and other nonspecified organs and tissues. Kidney EE was evaluated using equation 9. Other nonspecified organs and tissues EE was determined using equation 9 with the parameters of the “residual organs”. The adipose tissue EE was evaluated using the specific metabolic activity of fat mass proposed in a previous study (Guo and Hall 2011) (Appendix 4). Muscle EE was evaluated subtracting EEs of brain, heart, liver, GI, and “others” from the fat‐free mass (FFM) EE of the whole body. The EE of mouse and human FM and FFM are reported in Appendix 4. For human tissues/organs, the values of EE was evaluated using sum of the carbohydrate and fat substrate utilization rates estimated from flux balance analysis (Kim et al. 2007). The EE of mouse and human organs and tissues are reported in Table 4.

#### Mouse whole‐body VO_2_ prediction

**Figure 4.**
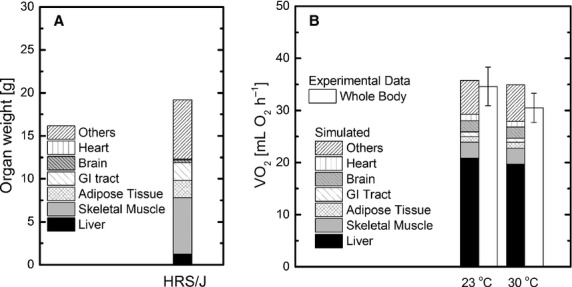
(A) Body composition; (B) Comparison of whole‐body VO_2_ of the HRS/J mouse strain between simulated and experimental data obtained at 23° and 30°C.

Using body composition and oxygen consumption data for each organ and tissue, the whole‐body VO_2_ (mL/h) can be predicted according to:
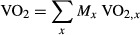


where *M*_*x*_ is the organ/tissue mass (g) and VO_2,*x*_ is the oxygen consumption (mL/h/g) of the organ/tissue *x* of a 30 g wild‐type mouse. For this prediction, the masses (Fig. 4A) and estimated rates of VO_2_ (Table 6) of mouse organs/tissues were used for HRS/J strain of mice at 23 and 30°C (Konarzewski and Diamond 1995).

#### Sensitivity analysis

We simulated the effect of ±25% changes of the metabolic fluxes and substrate uptake/release rates from mouse basal levels (Tables 1 and 2) derived from human data on carbohydrate and fat utilization rates. Some simulations of metabolic flux or substrate uptake/release variations produced negative intraorgan fluxes that were ignored as not physiological.

## Results

### Energy expenditure

The EE (per organ/tissue mass) of all mouse organs/tissues is significantly higher than their respective human organs/tissues (Table 4). The EE of brain, heart, liver, and GI tract in mouse is 3.0, 1.9, 7.8, and 1.4 times higher than the respective organ/tissue in human. The EE of muscle and adipose tissue in mouse are 6.0 and 24.5 times higher than those tissues in human. The EE of FFM and FM in mouse are 7.9 and 25.0 times higher than those tissues in human (Appendix 4).

### Organ/tissue metabolic fluxes and rates

For each organ/tissue, Equations (2–6) were solved to quantify metabolic fluxes and rates of O_2_ consumption, CO_2_ production, as well as rates of substrate uptake/release and utilization. As a representative case, the model calculation to estimate the liver metabolic fluxes is given in the Appendix 6 and results are presented in Figure A1. The inputs to the model equations are reported in the Tables 1–4 and 10. The solution of the mass and energy balance provides the rates of substrate uptake/release and gas exchange of mouse organs/tissues (Tables 5–6) and whole body (Table 7), the metabolic fluxes (Table 8), and substrate utilization (Table 9). For convenience, the simulated metabolic pathway fluxes are reported in Table 8 with the input metabolic fluxes highlighted in bold.

### Glucose uptake and gas exchange rates

The estimated glucose uptake in the brain and adipose tissue is within the range of the measured glucose uptake. The estimated glucose uptake, for heart is almost twofold higher, and for GI tract is an order‐of‐magnitude lower, than the measured glucose uptake (See Table 5). The VO_2_ and VCO_2_ are compared with those calculated with the standard approach Eq. 9 (Tang et al. 2002) (Table 6). The VO_2_ and VCO_2_ for brain, heart, GI tract, muscle, and adipose tissue estimated using these two approaches are similar. In contrast, the VO_2_ and VCO_2_ of the liver differ significantly between these two approaches.

### Whole‐body metabolic fluxes

With our methods, we could estimate whole‐body metabolic fluxes including glycogenolysis and gluconeogenesis (Table 7). The equations that relate organ/tissue to whole‐body metabolic fluxes are provided in Appendix 5. The results quantify the higher whole‐body metabolic fluxes in mouse compared to human. Gluconeogenesis in mouse is about 11 times higher than that in human. The rates of *de novo* lipogenesis, proteolysis, and lipolysis are about 16, 11, and 9 times respectively higher in mouse than those in human.

### Substrate utilization rates

The rates of carbohydrate (CHO) and FAT utilization in mouse organs/tissues are higher compared to human (Table 9). FAT utilization is absent in the brain and GI tract of mouse and human. The relative contributions of CHO and FAT to energy production are reported in Table 12. The percent contribution of CHO and FAT to energy production in the liver is significantly different for mouse and human, but they are only slightly different in heart, muscle, and adipose tissue. CHO is the only fuel for brain and GI tract energy metabolism and FAT utilization is absent in these organs. The negative carbohydrate utilization of liver indicates that liver is producing glucose with the energy from fat metabolism.

**Table 12.** Contribution of CHO and FAT oxidation to substrate utilization in mouse and human organs.

Organ/Tissue	CHO (%)		FAT (%)	
	Mouse	Human	Mouse	Human
Brain	100.0	100.0	0.0	0.0
Heart	30.5	30.8	69.5	69.2
Liver	−5.8	−18.1	105.8	118.1
GI tract	100.0	100.0	0.0	0.0
Skeletal muscle	36.6	33.4	63.4	66.6
Adipose tissue	30.9	30.7	69.1	69.3

### Whole‐body VO_2_ prediction

The predicted organ and whole‐body VO_2_ at 23°C and 30°C for HRS/J strain of mice were reported in Figure 4B. The simulated whole‐body VO_2_ is close to the experimental value at 23°C, while the simulated VO_2_ at 30°C is slightly higher than the measured value (Konarzewski and Diamond 1995).

### Sensitivity analysis

**Figure 5.**
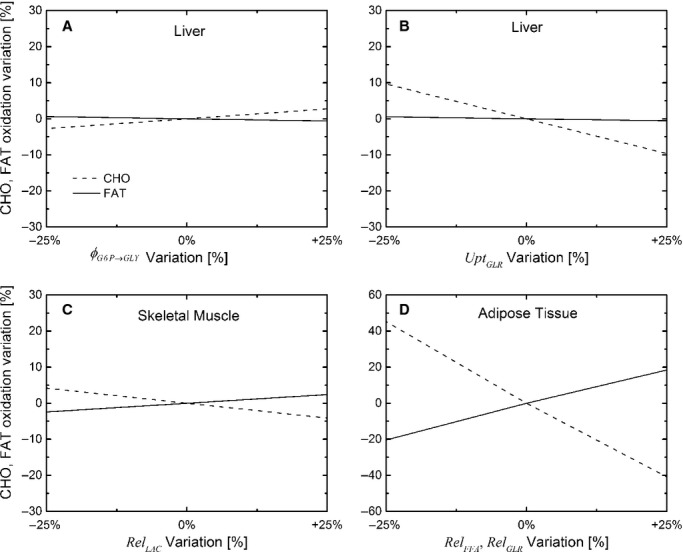
Sensitivity analysis. The effect of variation (±25% from the base case value) of *ϕ*_G6P→GLY_ in liver (A), Upt_GLR_ in liver (B), Rel_LAC__*,*_ in skeletal muscle (C), and simultaneous variation (±25% from the base case value) of *Rel*_FFA_ and Rel_GLR_ in adipose tissue (D) on carbohydrate and fat utilization.

The sensitivity results are presented in Figure 5. When *ϕ*_G6P→GLY_ flux in liver varied by ±25%, the carbohydrate utilization value varied slightly (±2.7%) and the fat utilization varied less than ±1% (Fig. 5A). From a ±25% variation of Upt_GLR,Liver_, the carbohydrate utilization changed by ±9.6%, and the fat utilization varied less than ±1% (Fig. 5B). When Rel_LAC,SM_ varied by ±25%, small changes occurred in carbohydrate (±4.1%) and fat utilization (±2.4%) (Fig. 5C). The variation in other metabolic fluxes and substrate uptake/release (±25%) derived from human data (Tables 1 and 2) had negligible (<1%) effect on carbohydrate and fat utilization rates. When *ϕ*_FFA*→*TG_ flux and Rel_FFA_ and Rel_GLR_ rates in adipose tissue were changed by ±25%, the results produced negative values of metabolic fluxes, which are not physiological. The simultaneous (+25%) variation of Rel_FFA_ and Rel_GLR_ affected carbohydrate and fat utilization rates by +20% and −40%, respectively (Fig. 5D).

## Discussion

### Mouse metabolism

In this study, a multiorgan analysis is applied to obtain mouse organ/tissue metabolic fluxes and rates of exchange of substrates. Using mass and energy balances for each organ, the rates of organ/tissue carbohydrate and fat utilization are evaluated. In turn, rates of substrate utilization were used to obtain organ/tissue energy expenditure (EE). The whole‐body and organ/tissue physiological parameters, EE, rates of substrate utilization, and rates of oxygen consumption of mice and humans are compared to quantify the differences in their energy and metabolic processes.

### Flux balance analysis in determining energy expenditure

The flux balance analysis with limited experimental data (organ/tissue metabolic fluxes and whole‐body metabolic parameters) can be used to quantify the fluxes that cannot be obtained easily with experiments and identifies the number of experiments required for obtaining unknown measurements (Tables 5,7–8). Currently, experimental data related to rates of free fatty acid uptake of heart, liver, skeletal muscle, lactate uptake/release of liver and adipose tissue, and glycerol release from GI tract are not available for mice. Our model analysis yields estimates of these rates. Furthermore, the model also estimates rates of glucose uptake for brain, heart, adipose tissue, and GI tract. We observed few differences between rates of glucose uptake obtained with model simulations and experiments. The rates of glucose uptake of brain and adipose tissue derived with model simulations are consistent with the experimental data (Table 5). This indicates that the model proposed with EE values and other assumptions utilized for these organs are consistent with experimental data obtained under similar physiological conditions. On the other hand, significant differences were noticed between estimated and measured rates of glucose uptake for heart and GI tract (Table 5). These differences in glucose uptake could be related to the inputs values for EE used for heart and GI tract. It is expected that for the same RQ, the increase in the EE of organs/tissues results in the simultaneous increase in the rates of carbohydrate and fat oxidation, which is followed by an increase in glucose and fatty acid uptake and vice versa. Therefore, the lower (or higher) glucose uptake for GI tract (or heart) can be caused by an underestimation (or overestimation) of the EE determined by allometric functions. This was verified by simulations using different EE values of GI tract and heart. The actual EE of GI tract and heart used for the FBA are 0.14 and 0.23 kcal/day, respectively. It was found that at 0.61 kcal/day EE value, the GI tract measured, and estimated rates of glucose uptake (234.3 *μ*mol/min/kg) are the same. At 0.2 kcal/day EE value, the measured and calculated rates of glucose uptake by the heart (49.1 *μ*mol/min/kg) are the same and the free fatty acid uptake by the heart decreased from 268.8 to 234.3 *μ*mol/min/kg (Table 5).

### Sensitivity analysis for model justification and experiment design

Under the assumption that the metabolic flux per unit organ/tissue mass in mouse and human are similar (Table 1), sensitivity analysis indicates that variations of most metabolic fluxes have a minor effect on the organ substrate utilization. Under the assumption that the appearance rate fractions of metabolic fuels in organs/tissues are similar in both human and mouse (Table 2), variations in all organ/tissue substrate uptake/release rates, only a few showed moderate sensitivity to carbohydrate and fat utilization. (Fig. 5B and C). Since FFA and GLR are both stoichiometrically related to lipolysis (3:1), Rel_FFA_ and Rel_GLR_ rates (Table 2) are closely coupled and significantly affect carbohydrate and fat utilization rates. Therefore, the relationship between Rel_FFA_ and Rel_GLR_ in mouse is similar in human. Since Rel_FFA_ and Rel_GLR_ were estimated using appearance rates of *R*_*a,*FFA_ and *R*_*a,*GLR_ from mouse, the substrate utilization rates estimated in the base case (Table 9 and 12) are plausible. Variation of most assumptions in Tables 1 and 2 has minimal effects on estimates of organ and whole‐body substrate utilization. This sensitivity analysis not only quantified the effect of assumptions on the model outputs, but also identified the most critical metabolic fluxes affecting organ substrate utilization and energy expenditure.

### Whole‐body metabolic fluxes

The model also yields estimates of whole‐body metabolic fluxes including gluconeogenesis, *de novo* lipogenesis, glycogenolysis, lipolysis, proteolysis, and oxidation of macronutrients (Tables 7, 9, and 12). These fluxes are higher in mouse than those in human organs/tissues. For example, the rates of gluconeogenesis and glycogenolysis in mouse liver are 11.4 and 2.7 fold higher than that in human liver, respectively. This is mainly due to the difference in the utilization of glucose as the fuel under overnight fasting conditions. This is supported by a glucose level in mouse plasma 6.6‐fold higher than that in human. The major source of glucose production under fasting conditions via gluconeogenesis and glycogenolysis is the liver.

### Comparison of mouse and human metabolism

The whole‐body energy expenditure (expressed per unit BW) in mouse is significantly higher than in human. Furthermore, the organ/tissue contribution to the whole‐body metabolic rate differs in mouse and human. The liver consumes about 52% and 20% of whole‐body energy expenditure in mouse and human, respectively, whereas the contributions of brain, heart, GI tract, skeletal muscle to the whole‐body energy expenditure in mouse are comparatively smaller than those in human (Table 4). These differences in the energy expenditure of mice and humans can be related to differences in the body composition and organ/tissue metabolic activities. While the size of liver and GI tract in mouse relative to body weight are about 3 times that in human, the proportions of skeletal muscle and adipose are lower in mice than that in human (Table 3). The energy expenditure of organs and tissues are higher in mouse than in human (Table 4), which can be related to the differences in the cellular and structural constituents of organs and tissues in these species. Although no direct evidence supports this argument, it can be inferred from studies (Elia 1992) that the energy expenditure of rat cerebral tissue is twofold higher than that in human. The higher energy expenditure of rat cerebral tissue was linked to a much smaller proportion of glial cells (i.e., lower energy expenditure). Furthermore, fiber type and composition of skeletal muscle vary across species. Similar muscles in different species may have different functional and metabolic properties (Schiaffino and Reggiani 2011; Bloemberg and Quadrilatero 2012). The citrate synthase activity, an indicator of mitochondria content, is higher in mouse than in human skeletal muscle, while the fraction of type I fibers in human skeletal muscle is higher than in rodents (Schiaffino and Reggiani 2011). Thus, the higher energy expenditure in mouse can be attributed to the higher mitochondrial density. The higher energy expenditure of mouse at whole‐body and organ/tissue levels is also related to higher rates of organ/tissue carbohydrate and fat oxidation (Table 9) and higher rates of glycogenolysis, gluconeogenesis, *de novo* lipogenesis, proteolysis, and lipolysis whole‐body metabolic fluxes (Table 7). The higher metabolic activity is also related to more heat loss in mouse than in human (Blaxter 1989).

### Mouse oxygen consumption rate

The model predicted the whole‐body VO_2_ at 23°C for HRS/J strain (Fig. 4B), but overestimated VO_2_ at 30°C by 15%. This may be related to data at ambient temperature, which can have a significant effect on the mouse metabolic rate (Speakman 2013). A temperature variation of 7–10°C leads to 10–30% of change of the basal metabolic rate (Konarzewski and Diamond 1995; Golozoubova et al. 2004) Therefore, an overestimation of the basal metabolic rate of 15% appears plausible since the model does not take into account the effect of the temperature on the energy expenditure.

The liver VO_2_ from flux balance analysis differ from indirect calorimetry (Table 6). This difference is mainly due to the inclusion of the stoichiometric reactions of glycogenolysis, gluconeogenesis from alanine and glycerol used for quantifying EE from the main metabolic pathways.

### Overview of model analysis

In this study, the organ/tissue contributions to the energy expenditure are quantified using a system of mass and energy balance equations based on the fluxes of the main energy metabolism pathways of each organ. This analysis incorporates available data on metabolic fluxes, substrate uptake and release rates, respiratory quotient (Tables 1–3), and organ/tissue EE allometric relationships. Since experimental data in support of various assumptions are lacking, the reliability of the model predictions is limited. On the other hand, this model analysis can be applied to identify the minimal set of metabolic flux measurements to determine the organ/tissues EE without using any assumptions for EE, metabolic fluxes, or respiratory quotients in Tables 1–3.

## Conclusions

The methodology developed in this study can be useful in the design of experimental studies to quantify the metabolic fluxes affecting energy expenditure in mouse models of disease. Furthermore, an integrative approach that combines limited experimental data and computational modeling can quantify changes in the tissue/organ metabolic activities taking into account body composition and metabolic or physiological differences between species. In future studies, contributions of kidney, lungs, and skin to the whole‐body energy balance can be included when sufficient data becomes available. To analyze weight regulation in disease, diet, or exercise, the tissue/organ metabolic flux network presented here would have to be integrated with hormonal control. In summary, the method presented quantifies the energy expenditure of mouse organs using metabolic flux measurements. This methodology can be used as an alternative approach to the traditional measurements based on Fick's principle to determine the organ energy expenditure. The theoretical framework is a paradigm for direct and quantitative human–mouse comparison of fuel utilization in tissue/organ systems and whole‐body fluxes under various metabolic or physiological conditions.

## Conflict of Interest

None declared.

## Figures and Tables

**Figure 8.**
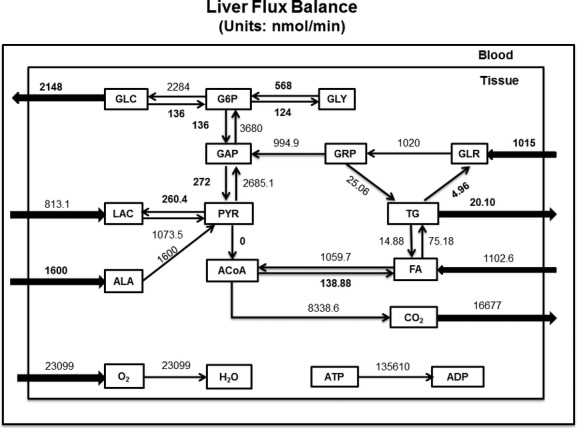
The model equations were solved using the function fsolve in MATLAB. Some of the data inputs were highlighted in bold font in the flux balance diagram. The other data inputs are, RQ: 0.72; EE: 225.7 10^−5 ^kcal min^−1^; CE^CHO^: 16.8 10^−9 ^kcal nmol^−1^; CE^FAT^: 16.6 10^−9 ^kcal nmol^−1^; All metabolic fluxes are in nmol min^−1^.

## Appendix

### Biochemical reactions of the metabolic pathways in tissue/organ *x*

**Table:**

1. Glycolysis I	GLC + ATP → G6P + ADP
2. Glycolysis II	G6P + ATP → 2GAP + ADP
3. Glycolysis III	GAP + Pi + NAD^+^ + 2ADP → PYR + NADH + 2ATP
4. Gluconeogenesis I	PYR + 3ATP + NADH → GAP + 3ADP + NAD^+^ + 2P_i_
5. Gluconeogenesis II	2GAP → G6P + P_i_
6. Gluconeogenesis III	G6P → GLC + P_i_
7. Glycogenesis	G6P + ATP → GLY + ADP + 2Pi
8. Glycogenolysis	GLY + Pi → G6P
9. Pyruvate reduction	PYR + NADH → LAC + NAD^+^
10. Lactate oxidation	LAC + NAD^+^ → PYR + NADH
11. Glycerol phosphorylation	GLR + ATP → GRP + ADP
12. GAP reduction	GAP + NADH → GRP + NAD^+^
13. Glycerol 3‐P oxidation	GRP + NAD^+^ → GAP + NADH
14. Alanine formation	PYR → ALA
15. Alanine utilization	ALA → PYR
16. Pyruvate oxidation	PYR + CoA + NAD^+^ → ACoA + NADH + CO_2_
17. Fatty acid oxidation	FA + 8CoA + 2ATP + 14NAD^+^ → 8ACoA + 2ADP + 2Pi + 14NADH
18. Fatty acid synthesis	8*A*CoA + 7ATP + 14NADH → FA + 8CoA + 7ADP + 7Pi + 14NAD^+^
19. Lipolysis	TGL → GLR + 3FA
20. Triglyceride synthesis	GRP + 3FA + 6ATP → TGL + 6ADP + 7Pi
21. TCA cycle	*A*CoA + ADP + Pi + 4NAD^+^ → 2CO_2_ + CoA + ATP + 4NADH
22. Oxidative phosphorylation	O_2_ + 6ADP + 6Pi + 2NADH → 2H_2_O + 6ATP + 2NAD^+^
23. Protein breakdown	Protein → ALA
24. ATP hydrolysis	ATP → ADP + Pi

## Appendix

### Steady‐state mass balance equations of metabolite in tissue/organ *x*

**Table:**

1. Glucose	*ϕ*_G6P→GLC_ − *ϕ*_GLC→G6P_ + *Q*_*x*_(*C*_*a*,GLC_ − *C*_*v*,GLC_) = 0
2. Pyruvate	*ϕ*_GAP→PYR_ + *ϕ*_LAC→PYR_ + *ϕ*_ALA→PYR_ − *ϕ*_PYR→GAP_ − *ϕ*_PYR→LAC_ − *ϕ*_PYR→ALA_ − *ϕ*_PYR→ACoA_ + *Q*_*x*_(*C*_*a*,PYR_ − *C*_*v*,PYR_) = 0
3. Lactate	*ϕ*_PYR→LAC_ − *ϕ*_LAC→PYR_ + *Q*_*x*_(*C*_*a*,LAC_ − *C*_*v*,LAC_) = 0
4. Alanine	*ϕ*_PYR→ALA_ + *ϕ*_Protein→ALA_ − *ϕ*_ALA→PYR_ + *Q*_*x*_(*C*_*a*,ALA_ − *C*_*v*,ALA_) = 0
5. Glycerol	*ϕ*_TG→GLR_ − *ϕ*_GLR→GRP_ + *Q*_*x*_(*C*_*a,*GLR_ − *C*_*v,*GLR_) = 0
6. Free Fatty acid	
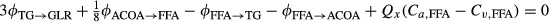
7. Triglyceride	
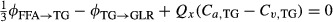
8. Oxygen	
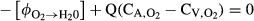
9. Carbon dioxide	
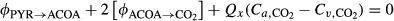
10. Glucose 6 phosphate	
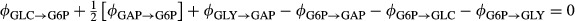
11. Glycogen	*ϕ*_G6P→GLY_ − *ϕ*_GLY→G6P_ = 0
12. Glyceraldehyde Phosphate	
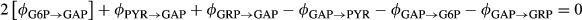
13. Glycerol phosphate	
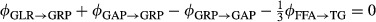
14. Acetyl coenzyme A	
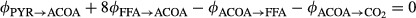
15. Coenzyme A	
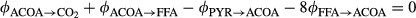
16. NAD+	
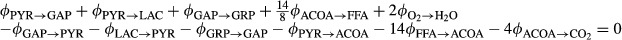
17. NADH	
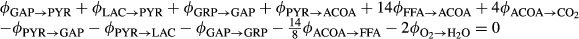
18. ATP	
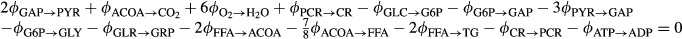
19. ADP	
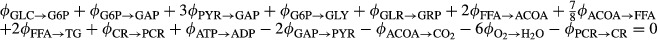
20. Pi	
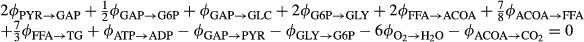

## Appendix

### Energy balance equations

**Table:**

Organ/Tissue	Carbohydrate utilization,
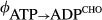
Brain	
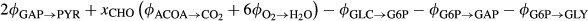
Heart	
Liver	
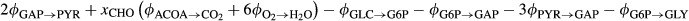
GI tract	
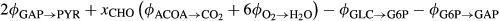
Skeletal muscle	
Adipose tissue

**Table:**

	Fat utilization
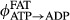
Brain	
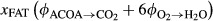
Heart	
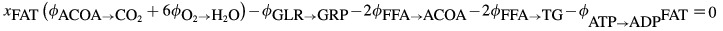
Liver	
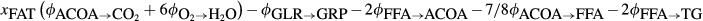
GI tract	
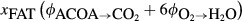
Skeletal muscle	
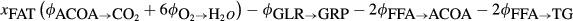
Adipose tissue	
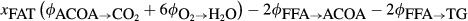

Where carbohydrate and fat oxidation fraction is defined as follows:

The overall energy balance for each organ/tissue system is

where, CE^CHO^ (16.825 10^−9 ^kcal/nmol) and CE^FAT^ 16.653 10^−9 ^kcal/nmol) are the carbohydrate and fat calorific equivalent of ATP, respectively.

## Appendix

### Estimation of FM and FFM energy expenditure

Under fasting conditions, the energy expenditure (EE) model for mouse reported by Guo and Hall (2011) reduces to

Where *K* is the basal thermogenesis rate, while *γ*_FM_ (30 kcal/kg/day) and *γ*_FFM_ (150 kcal/kg/day) are the specific metabolic rates of fat mass (FM) and free fat mass (FFM), respectively. The EE of FM and FFM are calculated with the following equations:

where *k* (per unit of body mass) is added to the metabolic rates of FFM and FM. We assumed that each gram of 30 g mouse equally contributes to basal thermogenesis. Therefore, *k* (K per unit of body mass) is 70 kcal/kg/day. The energy expenditure of FFM and FM are reported in Table 4.1.

**Table:**

**Table 4.1.** Energy Expenditure of fat and fat‐free mass of mouse and human.			
	EE (kcal/kg/day)^1^		*X*_*i*_‐fold
	Mouse (Guo and Hall 2011)	Human (Kim et al. 2007)	Mouse/Human
Fat‐free mass (FFM)	220.0	28.0	7.8
Fat mass (FM)	100.0	4.0	25.0

Human FFM EE (per unit FFM mass) was obtained by dividing sum of the absolute EEs of brain, heart, liver, GI tract, muscle, and others with their total mass and FM EE (per unit FM mass) is similar to the adipose tissue EE (per unit adipose mass).

## Appendix

### Whole‐body metabolic fluxes

**Table:**

1.	Glycogenolysis = where *i* is brain, heart, liver, GI, muscle, and adipose
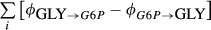
2.	Gluconeogenesis =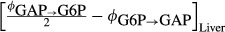
3.	De novo lipogenesis where *i* is brain, heart, liver, GI, muscle, and adipose
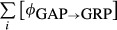
4.	Proteolysis = where *i* is brain, heart, liver, GI, muscle, and adipose
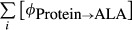
5.	Lipolysis = where *i* is brain, heart, liver, GI, muscle, and adipose
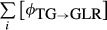

## Appendix

### Model calculations for quantifying liver metabolic fluxes

No of equations: (21) Mass balance (19), energy balance (1), congruence relationship (1)

No of variables: (38) RQ, EE, CE^CHO^, CE^FAT^, Rel_GLC_, Rel_TG_, Upt_GLR_, Upt_ALA_, Upt_LAC_, Upt_FFA_, , ,

Number of data inputs: (38−21 = 17) RQ, EE, CE^CHO^, CE^FAT^, Rel_GLC_, Rel_TG_, Upt_GLR_, Upt_ALA_,

**Table:**

Substrate steady‐state mass and energy balance equations (21)	
1. Glucose	
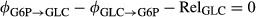
2. Glucose‐6‐phosphate	
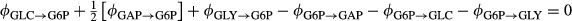
3. Glyceraldehyde phosphate	
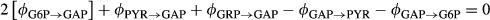
4. Lactate	*ϕ*_PYR→LAC_ − *ϕ*_LAC→PYR_ + Upt_LAC_ = 0
5. Pyruvate	*ϕ*_GAP→PYR_ + *ϕ*_LAC→PYR_ + *ϕ*_ALA→PYR_ − *ϕ*_PYR→GAP_ − *ϕ*_PYR→LAC_ − *ϕ*_PYR→ACoA_ = 0
6. Alanine	− *ϕ*_ALA→PYR_ + Upt_ALA_ = 0
7. Triglyceride	
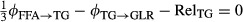
8. Glycerol	*ϕ*_TG→GLR_ − *ϕ*_GLR→GRP_ + Upt_GLR_ = 0
9. Glycerol phosphate	
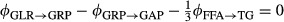
10. TG to FFA	*ϕ*_TG→FFA_ − 3*ϕ*_TG→GLR_ = 0
11. GRP to TG	
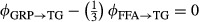
12. Free fatty acid	
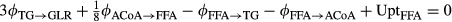
13. Acetyl coenzyme A	
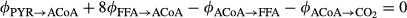
14. Oxygen	
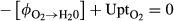
15. Carbon dioxide	
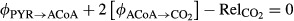
16. Respiratory quotient	
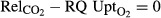
17. ATP	
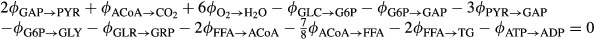
18. CHO utilization	
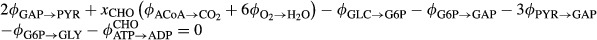
19. FAT utilization	
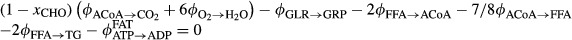
20. CHO contribution to TCA cycle	
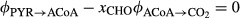
21. Overall energy balance	
